# Microcirculation in the conjunctiva and retina in healthy subjects

**DOI:** 10.1186/s40662-019-0136-3

**Published:** 2019-04-06

**Authors:** Ce Shi, Hong Jiang, Giovana Rosa Gameiro, Jianhua Wang

**Affiliations:** 1 School of Ophthalmology and Optometry, Wenzhou Medical University, Wenzhou, Zhejiang China; 2 Bascom Palmer Eye Institute, University of Miami, Miller School of Medicine, 1638 NW 10th Avenue, McKnight Building - Room 202A, Miami, FL 33136 USA

**Keywords:** Variability, Bulbar conjunctiva, Blood flow velocity, Microvascular network, Functional slit-lamp biomicroscopy (FSLB), Hemodynamics, Microcirculation, Retina, Conjunctiva, Retinal function imager

## Abstract

**Background:**

The aim was to determine the relationship between bulbar conjunctival microcirculation and retinal microcirculation in a healthy population.

**Method:**

A functional slit-lamp biomicroscope (FSLB) was used to measure blood flow velocity (BFV) and blood flow rate (BFR) in the conjunctiva while a retinal function imager (RFI) was used to measure macular BFV and BFR in the retina. One eye of each subject of 58 self-reported healthy subjects was imaged in the same session on the same day.

**Results:**

The mean BFV in the venules of the conjunctiva was 0.49 ± 0.13 mm/s, which was significantly slower than that in the retinal arterioles (3.71 ± 0.78 mm/s, *P* < 0.001) and retinal venules (2.98 ± 0.58 mm/s, *P* < 0.001). The BFR in the conjunctiva (0.09 nl/s) was also significantly lower than that in the retina (arterioles = 0.81 nl/s, venules = 0.68 nl/s, all *P* < 0.001). The BFVs and BFRs were not related between the conjunctiva and retina (r ranged from − 0.17 to − 0.05, all *P* > 0.05).

**Conclusion:**

The microcirculation in the retina appeared to be different from that in the conjunctiva.

## Background

The eye and the brain share similar embryological origins, with similar structural and physiological characteristics. The eye is regarded as a window to the brain and helps monitor microcirculation and neurodegeneration. The conjunctival and retinal microvessels both originate from the internal carotid artery, and both include capillaries and pre-capillary arterioles and post-capillary venules. Using current techniques of ophthalmic imaging such as the functional slit-lamp microscopy (FSLB) [1–3] and retinal function imager (RFI) [4, 5], the microcirculation of the conjunctiva and retina can be measured, which may help improve the current understanding of the characteristics of the microvasculature and microcirculation in the normal population during aging and under disease conditions.

Our previous studies using advanced ophthalmic imaging on bulbar conjunctival microvasculature and microcirculation have indicated that the microcirculation of the conjunctiva declines with age in healthy subjects [1]. Short-term [2] and longitudinal contact lens wear [3] affected the microcirculation in the conjunctiva. Similarly, the microcirculation measured as retinal blood flow velocity (BFV) in the retina also declines with age in healthy subjects [4]. Impaired retinal microcirculation have been found in multiple sclerosis [5] and Alzheimer’s disease [6]. An intriguing phenomenon is that the BFVmeasured in the post-capillary venules in the retina [4, 5] with similar diameters as the conjunctival vessels is about ten times faster than the venules in the conjunctiva [1–3]. Although the BFVmeasured in the retina was higher than that in the conjunctiva, these measurements were not evaluated in the same subjects. Furthermore, it remains unknown whether the blood flow velocities measured in the conjunctiva and retina are related. This study aimed to determine the relationship between the microcirculation in the conjunctiva and the retina in the same subjects from a healthy normal population.

## Methods

The study was approved by the ethics committee board of the University of Miami and conducted according to the tenets of the Declaration of Helsinki. All subjects were recruited and informed about the purposes and methods of the study and each volunteer signed a consent form.

### Study subjects

A total of 58 normal healthy subjects were recruited at the Bascom Palmer Eye Institute of the University of Miami. All subjects with a refractive error of no more than − 6.00 D and no greater than + 3.00 D were recruited. Only one eye of each subject was selected to be imaged. All subjects were imaged within 1 hour at the same visit during office hours (from 9 AM to 5 PM) [7]. The exclusion criteria included a history of contact lens wear (within 6 months), ocular surgery and trauma, systemic diseases, and the use of medications. Subjects with hypertension, diabetes, sickle cell anemia, cerebral small vessel disease, history of stroke, cardiovascular diseases, and other vascular diseases were also excluded.

### Study examinations

All subjects underwent complete ophthalmologic examinations, including the slit-lamp biomicroscope, best-corrected visual acuity (BCVA) measurement, fundus examination, and intraocular pressure. The best-corrected visual acuity of all subjects was 20/20 or above. Their blood pressures, including systolic pressure and diastolic pressure, and heart rates were also measured, and personal medical histories were obtained from all subjects.

### Functional slit-lamp biomicroscope (FSLB) for measuring conjunctival microcirculation

**Fig. 1:**
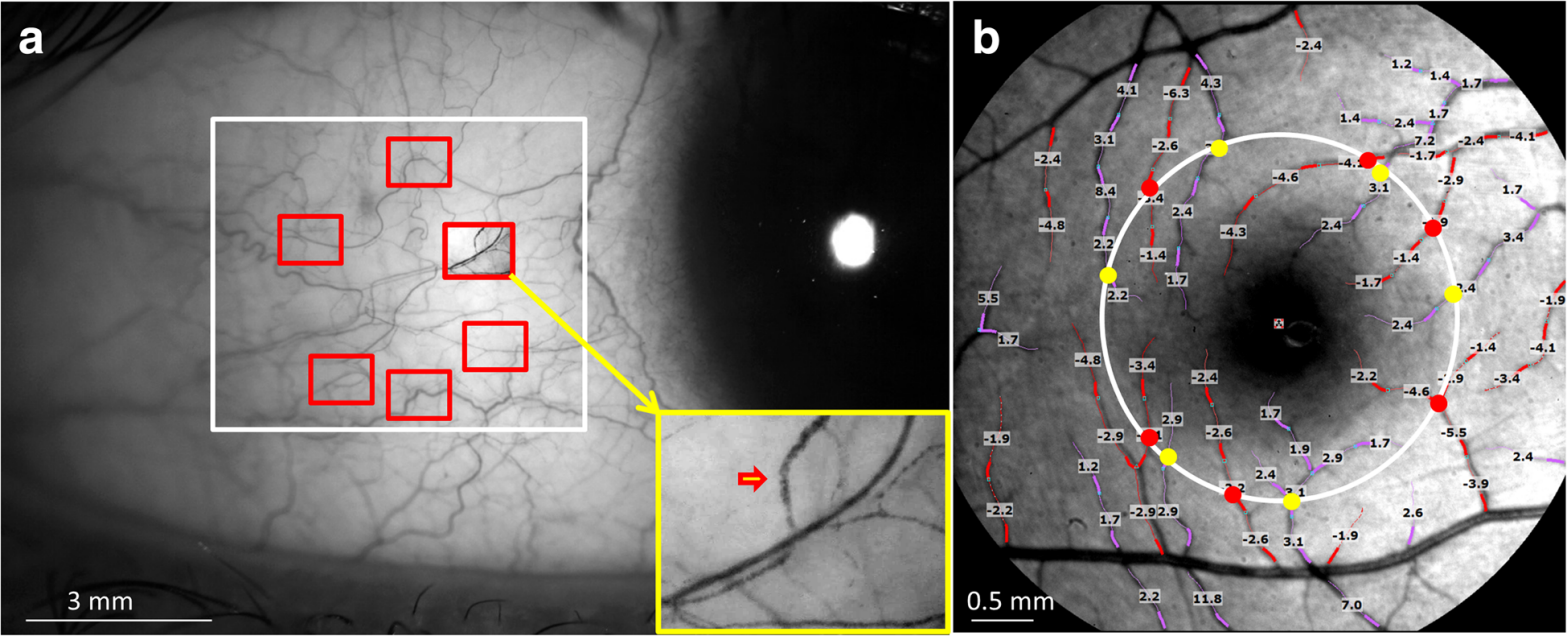
Blood flow velocity and blood flow rate of the conjunctiva and retina. FSLB and RFI were used to measure the blood flow velocity and flow rate in the conjunctiva (**a**) and retina (**b**). A total of six fields of view (red rectangles inside the white rectangle) were imaged using FSLB in the temporal side of the bulbar conjunctiva 1 mm apart from the limbus. With the extremely high magnification (~ 210 ×), the cluster of red blood cells was visualized (red arrowhead in the insert), which facilitated the measurement of blood flow velocity and flow rate. Retinal blood flow velocity was measured in the 2nd and 3rd branches of retinal arterioles (red) and venules (purple) which were overlaid with the measured blood flow velocity (note: negative values indicate arteriolar velocity). To estimate the blood flow in the retina, a circle (white, diameter = 2.5 mm) centered on the fovea was drawn. Vessel diameters of the vessels crossing the circle were measured in the arterioles (red dots) and venules (yellow dots). The velocity and vessel diameter were used to calculate the flow rate

FSLB settings and image procedures have been well described in previous studies [2, 8], and its repeatability had been validated [7]. In brief, the FSLB was modified from a traditional slit-lamp by adding a digital camera which has a special function called Movie Crop Function (MCF). The MCF enables the addition of a 7× magnification, which can be combined with the slit-lamp magnification (30×), resulting in extremely high magnification (~ 210×) for imaging the motion of the cluster of red blood cells (Fig. 1). In this study, the field of view of the FSLB was 0.9 × 0.7 mm. The pixel interval on the acquired video was 1.4 μm. The other settings of the camera were the same as in previous studies [7, 9]. To measure the mean BFV and blood flow rate (BFR), six different locations approximately 1 mm away from the limbus were recorded. The measurement was taken on conjunctival venules because the majority of the conjunctival vessels are venules. Custom software was used to obtain BFV and BFR and the detailed image processing procedures have been reported previously [7, 9].

### Retinal function imager (RFI) for measuring retinal microcirculation

RFI is an advanced ophthalmic imaging modality based on a fundus camera. The system and its applications have been recently reviewed [10] and the system has a high reproducibility [11]. A high-speed camera is attached to the fundus camera with a green illumination system to acquire a series of fundus photos. The image processing software processed the sequential images to obtain the motion of a cluster of red blood cells in the 2nd and 3rd branches of pre-capillary arterioles and post-capillary venules for the measurement of the mean retinal BFV (Fig. 1) [4, 5]. To measure the mean retinal BFR, a circle (diameter = 2.5 mm) was drawn to outline the vessels crossing the circle. The diameter of these vessels was measured. Using both the measured BFV and vessel diameter, BFR was calculated. In the present study, the field of view was 4.3 × 4.3 mm. The pixel interval of the captured image was 4.2 μm. Before imaging, 1% tropicamide was used to dilate the pupil [12].

### Statistical analysis

All data are presented in the format of mean ± standard deviation (SD) and analyzed using Excel (version 2010; Microsoft, Redmond, WA, USA). The sample size was calculated by a software program (G*Power, version 3.1.9.4) recommended by Faul et al. [13] and Bonett and Wright [14]. A sample size of 14 subjects would be enough to detect the difference of the microcirculation in the retina and conjunctiva with a detection power of 0.99. Paired student t-test was used to compare the microvascular parameters in the conjunctiva and retina. Pearson correlation coefficients were used to determine the relationships between the microvascular parameters in the conjunctiva and the retina. All *P* < 0.05 were regarded as statistically significant.

## Results

**Fig. 2:**
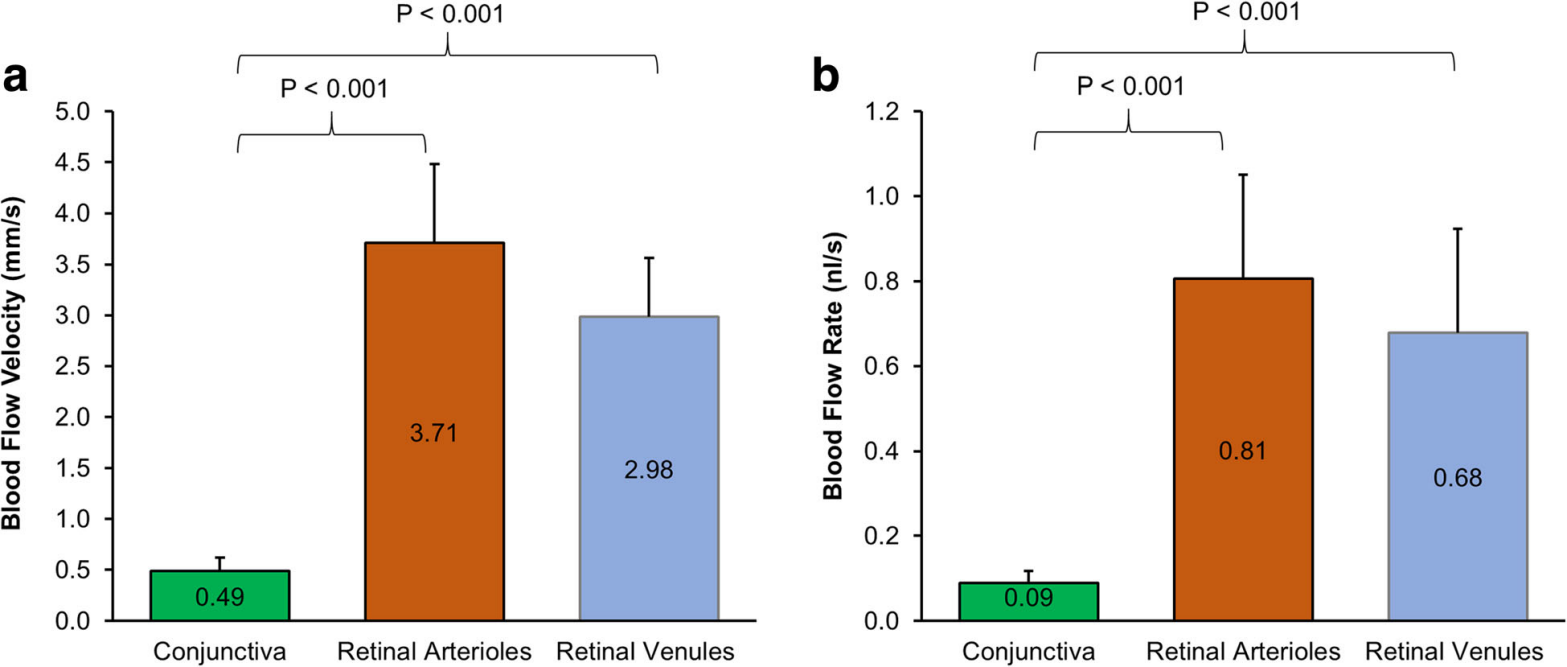
Comparison of conjunctival and retinal microcirculation. Blood flow velocity (**a**) and blood flow rate (**b**) were significantly lower in the conjunctiva compared to retinal arterioles and venules (all *P* < 0.001)

**Fig. 3:**
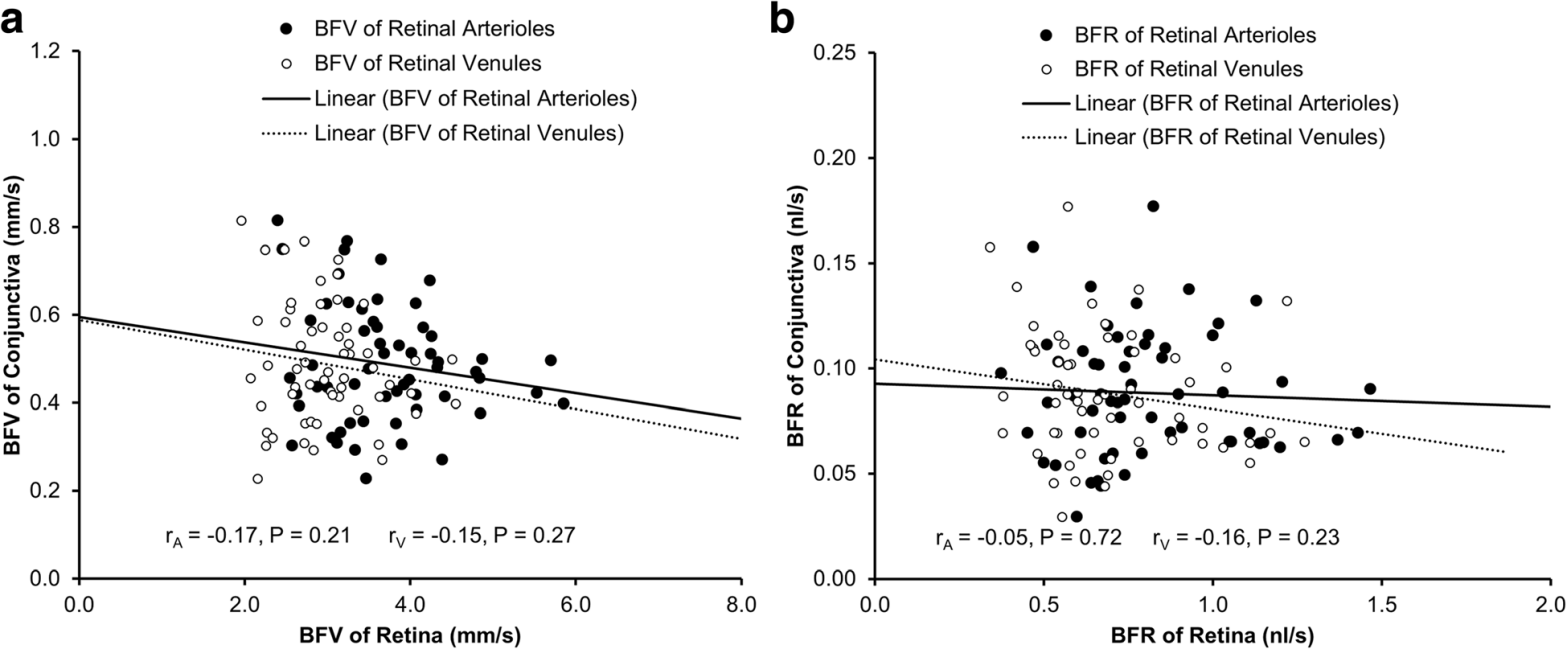
Correlations of conjunctival microcirculation with retinal microcirculation. Conjunctival blood flow velocity (BFV) had no significant correlations with the BFVs of the retinal arterioles (r = − 0.17, *P* = 0.21) and venules (r = − 0.15, *P* = 0.27, **a**). Likewise, conjunctival blood flow rate (BFR) had no significant correlations with the BFRs of the retinal arterioles (r = − 0.05, *P* = 0.72) and venules (r = − 0.16, *P* = 0.23, **b**)

Demographics and baseline characteristics of the healthy normal subjects are shown in Table 1. The mean BFV in the conjunctiva was 0.49 ± 0.13 mm/s, which was significantly slower than that in the retinal arterioles (3.71 ± 0.78 mm/s, *P* < 0.001, Fig. 2) and retinal venules (2.98 ± 0.58 mm/s, *P* < 0.001). The BFR in the conjunctiva was also significantly lower than that in the retina (*P* < 0.001, Fig. 1). The BFVs and BFRs were not related between the conjunctiva and retina (r ranged from − 0.17 to − 0.05, *P* > 0.05, Fig. 3).

**Table 1:** Demographics of study subjects

		Range
Number of subjects	58	
Male vs. Female	21: 37	
Age (years)	34.0 ± 9.2	17–56
Systolic Blood Pressure (mmHg)	112.8 ± 10.6	93–142
Diastolic Blood Pressure (mmHg)	73.7 ± 8.7	55–91
Heart Rate (bpm)	72.3 ± 10.0	52–95

Results are presented as mean ± standard deviation

**Fig. 4:**
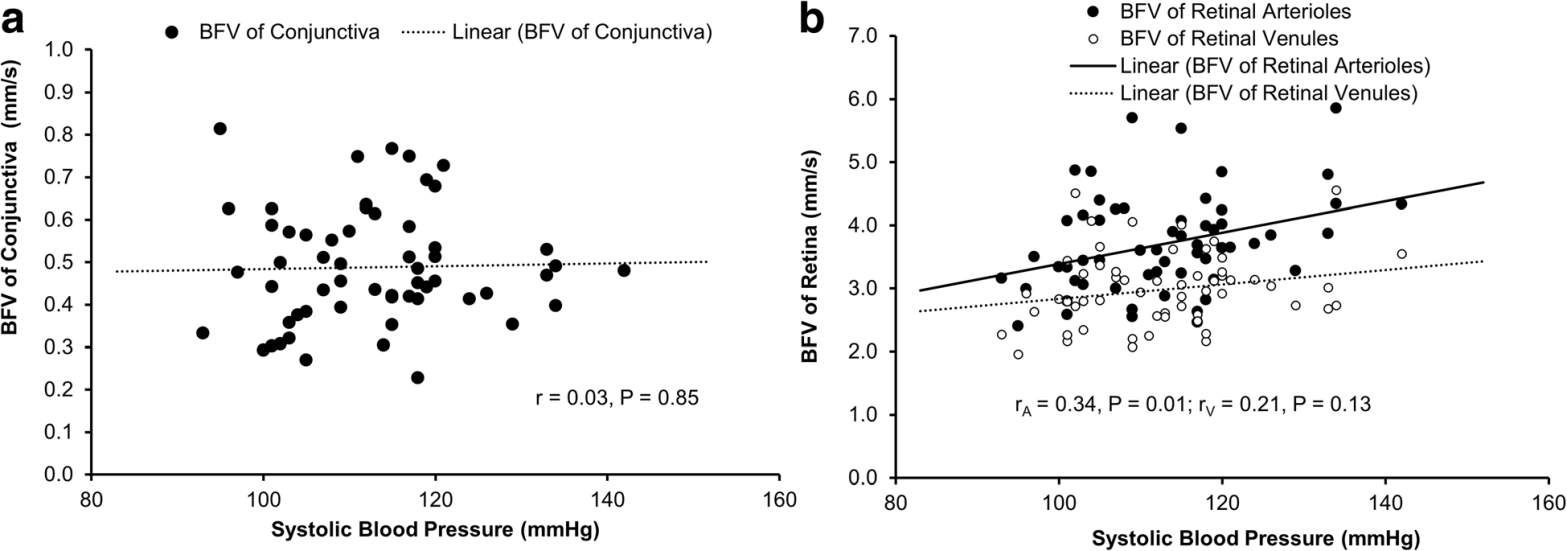
Correlations of systolic blood pressure with BFV of the retina and conjunctiva. The BFV of the conjunctiva had no significant correlation with systolic blood pressure (r = 0.03, *P* = 0.85, **a**). On the other hand, BFV of retinal arterioles was positively correlated with systolic blood pressure (r = 0.34, *P* = 0.01, **b**). No significant correlation was found between the BFV of retinal venules and systolic blood pressure (r = 0.21, *P* = 0.13, **b**)

The arteriolar and venular BFV in the retina had a significant positive correlation with age (r = 0.39, r = 0.29, respectively, both *P* < 0.05, Table 2). The arteriolar BFRs also had a significant positive correlation with age (r = 0.27, *P* = 0.04, Table 2). The arteriolar BFV in the retina had a significant positive correlation with systolic blood pressure (r = 0.34, *P* = 0.01, Fig. 4, Table 2). Other microvascular parameters in the conjunctiva and retina had no significant correlations with age, systolic blood pressure, diastolic blood pressure, and heart rate (r ranged from − 0.23 to 0.03, all *P* > 0.05).

**Table 2:** Correlations between the microcirculation of the conjunctiva and retina with systemic variables

		Age (years)	SBP (mmHg)	DBP (mmHg)	HR (bpm)
Retinal Microcirculation					
Arteriolar BFV (mm/s)	r	0.39	0.34	0.22	0.18
	P	0.003*	0.01*	0.10	0.19
Venular BFV (mm/s)	r	0.29	0.21	0.10	0.24
	P	0.03*	0.11	0.46	0.07
Arteriolar flow rate (ql/s)	r	0.27	0.17	0.10	0.003
	P	0.04*	0.21	0.45	0.98
Venular flow rate (ql/s)	r	0.19	0.16	0.06	−0.01
	P	0.15	0.24	0.68	0.94
Conjunctival Microcirculation					
BFV (mm/s)	r	−0.23	0.03	0.06	0.13
	P	0.08	0.85	0.64	0.35
Blood flow rate (ql/s)	r	−0.24	0.06	0.14	0.11
	P	0.07	0.64	0.29	0.42

*BFV* = blood flow velocity; *SBP* = systolic blood pressure; *DBP* = diastolic blood pressure; *HR* = heart rate; *P* values < 0.05 are denoted by an asterisk

## Discussion

This is the first study that demonstrates the relationship between the circulation in the conjunctiva and retina in the same subjects from a healthy normal population. The key finding was that the retinal microcirculation and the conjunctival microcirculation were different and not related. There may be a few reasons for this. First, the vessel diameter in the bulbar conjunctiva and retina was slightly different. The vessel diameter of the retinal vessels was about 20 μm [12] and that of the conjunctival vessels was about 16 μm [1]. Normally, larger vessels would have higher blood flow velocities [15]. However, the velocity in the retinal venules was ten times faster than that in the conjunctiva. Therefore, the difference in vessel diameters may only partially explain the differences in BFV and BFR. Second, the conjunctival venules are exposed to the outer environment, which can easily be affected by environmental factors such as temperature and humidity or by some ocular diseases such as dry eye [16]. In contrast, the retinal venules are well protected in the inner eye and have a more stable environment inside the eye when compared to the conjunctiva. Third, the conjunctiva and retina may play different roles in supplying oxygen and nutrition to the tissue. In the visual system, the retina, especially the macula, plays an important role in central visual acuity, which has a higher metabolic demand. A faster BFV and BFR could provide a quicker supply of oxygen and nutrition to the retina. In contrast to the retina, the conjunctiva serves as a protective membrane on the ocular surface. Low BFV and BFR may be sufficient for maintaining the function of the conjunctiva. Fourth, the retina lacks vegetative nerve stimulation [17] and its microcirculation is autoregulated [18] and also affected by the intraocular pressure pulse [19]. These features may contribute to the retinal microcirculation, which is very different than that in the other human organs, including the conjunctiva. Lastly, the difference between the microvascular network densities may also explain the difference in BFV and BFR. Wei et al. reported that the fractal dimension Dbox representing the vessel density in the retina of normal healthy subjects was 1.8 [4], which appears to be higher than that in the conjunctiva (Dbox = 1.6) [1] although no direct comparison was performed.

The microcirculation in the arterioles in the retina appeared to be affected by systemic variables such as blood pressure. A positive correlation was found between systolic blood pressure and arteriolar BFV in the retina. The arterioles branch out from an artery and lead to capillaries, which may be more influenced by the systolic blood pressure. On the other hand, both BFV measurements of the venules in the conjunctiva and retina were not associated with blood pressure, indicating that both venules in the conjunctiva and retina may share some common characteristics whether anatomical or otherwise. As we did not include patients with hypertension, further studies will be needed to validate our hypothesis. Interestingly, positive correlations were found between age and BFVs in the retina, which was not in agreement with a previous study from our group [4]. In that previous study conducted by Wei et al. [4], a group of 74 subjects (age range: 18 to 82 years) was imaged using RFI and a negative correlation between retinal venular velocity and age was observed. The relationship between retinal arteriolar BFV and age was positive but not significant (r = 0.09, *P* = 0.44). In contrast, a group of 58 healthy subjects (age range: 17 to 56 years) was studied using RFI in the present study. Positive relationships between retinal BFVs in the arterioles and venules and age were found. This discrepancy appears to be due to the different study groups with different age ranges.

Our study has some limitations. First, two different machines were used to access the microcirculation in the conjunctiva and retina because there was no single machine available that could access the microcirculation of both. Second, we did not image the microcirculation of the retina and conjunctiva simultaneously. Further studies using the same imaging device (if available) could overcome the drawback encountered in the present study. Third, although all subjects were imaged using both imaging devices within 1 h at the same visit during office hours, the diurnal changes during office hours may influence the comparison between the conjunctival and retinal vascular measurement; however, in our experience, this is unlikely. Nevertheless, further studies are needed to identify the impact of the diurnal changes on blood flow measurements and their relationship between the conjunctiva and retina. Fourth, the fields of view and pixel relationships are different between RFI and FSLB, depending on their cameras and magnifications. However, both systems are capable of tracking the movement of clusters of red blood cells so that the measurements of the BFV can be performed. Fifth, we only measured the venules in the conjunctiva and measured both arterioles and venules in the retina because the majority of the conjunctiva vessels are venules. We calculated the measurements of the venules as done previously [1, 9].

## Conclusions

In conclusion, this is the first study revealing that the microcirculation in the retina appears to be different in BFV and BFR from that in the conjunctiva, which may be due to the lack vegetative nerve stimulation, autoregulation and possible intraocular pressure pulse on the retina. Future studies with a diseased population may further reveal the nuances in the microcirculation in the conjunctiva and retina.
